# Space–time graded-index interfaces and related chirping

**DOI:** 10.1515/nanoph-2025-0308

**Published:** 2025-09-05

**Authors:** Zhiyu Li, Xikui Ma, Klaas De Kinder, Amir Bahrami, Christophe Caloz

**Affiliations:** Xi’an Jiaotong University, Xi’an, China; KU Leuven, Leuven, Belgium

**Keywords:** space–time modulation, graded-index media, generalized impulse response, instantaneous frequency, chirping

## Abstract

Space–time modulated systems have recently emerged as a powerful platform for dynamic electromagnetic processing in both space and time. Most of the related research so far has assumed abrupt parameter profiles. This paper extends the field to generalized graded-index (GRIN) interfaces, which are both more practical than ideal profiles and offer new avenues for wave manipulations. It presents an exact solution for wave propagation across arbitrary space–time modulated GRIN interfaces and describes versatile chirping effects. The solution is based on a generalization of the impulse response method from linear time-invariant to linear *space–time-varying* systems. The proposed framework shows that space–time GRIN systems represent a novel approach for generating a new form of chirping that is not inherently based on dispersion, with promising applications in pulse shaping and signal processing.

## 1 Introduction

Space–time modulation systems – media whose parameters vary dynamically in both space and time under the influence of an external traveling-wave modulation – have recently enabled a range of novel applications, including magnet-free nonreciprocity [1], frequency transitioning [2], parametric amplification [3], [4], and the breaking of fundamental bounds [5].

**Figure 1:**
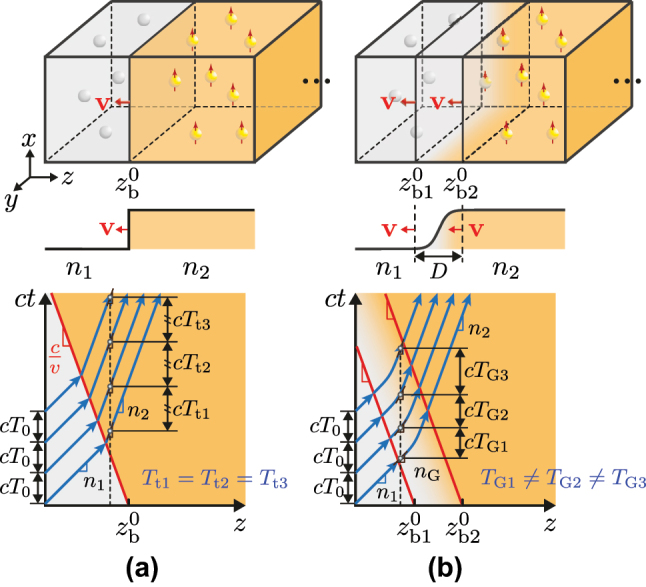
Space–time modulation interfaces, with basic structures (top) and space–time diagrams in the harmonic-wave regime (bottom). (a) Step-index modulation, where the refractive index changes abruptly from *n*_1_ to *n*_2_ at an interface initially located at $z_{\text{b}}^{0}$ and moving with a constant velocity **v**. (b) Graded-index (GRIN) modulation, where the refractive index traditions smoothly from *n*_1_ to *n*_2_ via *n*_G_ over the interval $\left[z_{\text{b}1}^{0},z_{\text{b}2}^{0}\right]$ with width $D=z_{\text{b}2}^{0}-z_{\text{b}1}^{0}$ . The subscripts *G* and *b* denote the GRIN layer and the boundary, respectively.

Space–time interfaces are the fundamental building blocks – or “meta-atoms” – of space–time modulation systems. Their understanding and control are, therefore, essential. These interfaces can be of two distinct types, as illustrated in Figure 1. The first is the *step-index interfaces*, where the transition width is much smaller than the wavelength, as shown in Figure 1(a). Such interfaces have been extensively studied in the literature [6], [7] and are known to induce a uniform frequency shifting of the incoming wave. The second type is the *graded-index (GRIN) interfaces*. These interfaces, shown in Figure 1(b), may be considered more practical, as physical modulations necessarily occur gradually at the microscopic level [8], [9], [10], and can also support a broader range of wave transformations. In particular, they induce nonuniform frequency transitions and produce wave chirping. While some studies have addressed the limiting case of pure-time GRIN interfaces [11], [12], [13], [14], general space–time GRIN interfaces [15] remain an essentially unexplored topic.

This paper investigates the wave transformations induced by space–time GRIN interfaces, introducing a generalization of the impulse response method from linear time-invariant (LTI) to linear (space-) time-varying (LTV) systems. Unlike the Wentzel–Kramers–Brillouin (WKB) [11], [12], [13] or transfer-matrix method (TMM) [15] commonly used in prior works, this approach provides an *exact* solution. We derive closed-form solutions for both the analysis and synthesis problems and describe the corresponding chirping effects. All the results are validated through full-wave simulations.

For simplicity, we restrict our attention to systems that are 1 + 1D, with dimensions *z* and *t*, involving GRINs of uniform velocity, i.e., $v=v\hat{z}=const.$ , boundary and intrinsic impedance matching, i.e., $\eta_{i}=\sqrt{\mu_{i}/\epsilon_{i}}=const.$ for *i* = 1, G, 2, and no dispersion, i.e., *n*_*i*_ ≠ *n*_*i*_(*ω*) for *i* = 1, G, 2.

## 2 Generalized impulse response method

**Figure 2:**
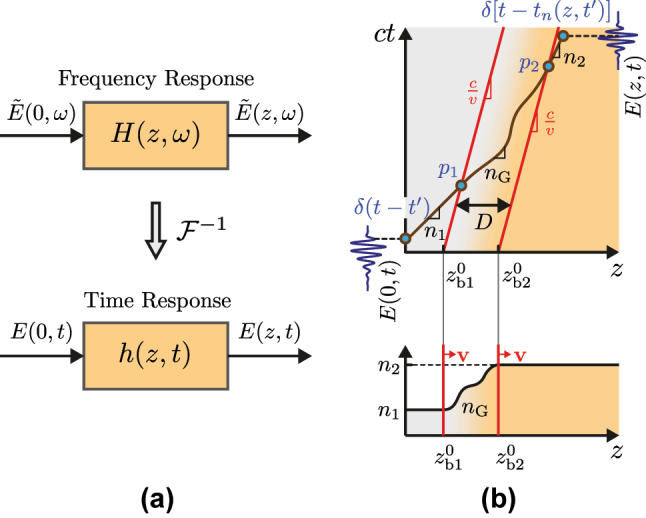
Generalized impulse response method applied to the space–time GRIN interface system in Figure 1(b). (a) Responses in the frequency and time domains. (b) Response of an arbitrary space–time GRIN modulation system. The top panel shows the space–time diagram with the trajectory of the impulse *δ*(*t* − *t*′) and its output $\delta \left[t-t_{n}\left(z,t^{\prime }\right)\right]$ , where *p*_1,2_ are the space–time intersection points of the impulse with the two boundaries of the GRIN layer, initially located at $z_{\text{b}1,2}^{0}$ . The bottom panel shows the initial refractive index profile *n*(*z*, *t* = 0), which moves at a uniform velocity **v**.

In this section, we shall derive the generalized impulse for the GRIN system in Figure 1(b), which is generically represented in Figure 2.

In the frequency domain, the one-dimensional Helmholtz equation may be expressed as

(1) $$\frac{\partial^{2}\tilde{E}}{\partial z^{2}}+k^{2}\left(\omega \right)\tilde{E}=0,$$

where $k\left(\omega \right)=\omega \sqrt{\mu \epsilon }=\omega n/c$ is the wavenumber. The general solution to Eq. (1) can be written as

(2a) $$\tilde{E}\left(z,\omega \right)=H\left(z,\omega \right)\tilde{E}\left(0,\omega \right),$$

where

(2b) $$H\left(z,\omega \right)=e^{\text{i}k\left(\omega \right)z}$$

represents the frequency response of the system.

Applying the temporal inverse Fourier transform to Eq. (2), as suggested in Figure 2(a), we obtain the corresponding time-domain relation

(3) $$E\left(z,t\right)=\overset{\infty }{\underset{-\infty }{\int }}h\left(z,t,t^{\prime }\right)E\left(0,t^{\prime }\right)\;dt^{\prime },$$

where *h*(*z*, *t*, *t*′) is the impulse response [16], representing the system’s response to the impulse *E*(0, *t*) = *δ*(*t* − *t*′).1The impulse response acts as a time-domain version of the Green’s function in the space-domain relation **E**(**r**) = i*ωμ∫*_*V*_ *G*(r, **r**′)**J**(**r**′) d*V*′ [17], with *E*(0, *t*′) in Eq. (3) playing the role of the current source **J**(**r**′) and *h*(*z*, *t*, *t*′) corresponding to the Green’s function *G*(**r**, **r**′).


In LTI systems, the impulse response depends solely on the time difference between the output and input signals, following the time-shift invariance property *h*(*z*, *t*, *t*′) = *h*(*z*, *t* − *t*′) [18]. Xiao et al. extended the impulse response method from LTI to purely time-varying systems [19], where the lack of time invariance – or nonstationarity – leads to an impulse response that independently depends on *t* and *t*′, i.e.,

(4) $$h\left(z,t,t^{\prime }\right)\neq h\left(z,t-t^{\prime }\right).$$

In space–time varying systems, *h*(*z*, *t*, *t*′) also involves the modulation velocity *v*, which further increases the complexity of the analysis. We now introduce the generalization of the impulse response method to space–time GRIN systems, explicitly incorporating *v*, with the aid of Figure 2(b), where a space–time diagram illustrates the impulse trajectory.

As shown in the top panel of Figure 2(b), the input impulse experiences a propagation delay as it propagates through the system, resulting in the impulse response

(5) $$h\left(z,t,t^{\prime }\right)=\delta \left[t-t_{n}\left(z,t^{\prime }\right)\right],$$

where *t*_*n*_(*z*, *t*′) is the arrival time of the impulse at the position *z* assuming an input time of *t*′ in the GRIN system with space–time varying refractive index *n* [brown line in the top panel of Figure 2(b)].2In a nondispersive system, the impulse response exhibits the form of a delta function as all the frequency components propagate at the same velocity, and the input impulse remains undistorted. In contrast, in dispersive systems, not considered here, the frequency-dependent group velocity causes temporal spreading, and the impulse response becomes a more complex function of *t*′ [16]. The function *t*_*n*_(*z*, *t*′) is determined by the wave trajectory equation

(6) $$c\frac{dt_{n}}{dz}=n\left(z,t_{n}\right),$$

subject to the boundary conditions in different regions

(7) $$t_{n}\left(0\right)=t^{\prime }\;\text{and}\;t_{n}\left(z_{\text{b}1,2}\right)=t_{\text{b}1,2},$$

where *z*_b1,2_ and *t*_b1,2_ are the coordinates of the intersection points *p*_1,2_ of the impulse with the first and second modulation interfaces, respectively [Figure 2(b)].

To derive the electromagnetic field in each region of the space–time GRIN system, we will apply the following four steps to each of the three regions in Figure 2(b): (i) determine the trajectory equation and corresponding boundary condition for the concerned region; (ii) solve the trajectory equation for the arrival time *t*_*n*_(*z*, *t*′); (iii) substitute the resulting *t*_*n*_(*z*) into Eq. (5) to obtain the impulse response *h*(*z*, *t*, *t*′); and (iv) substitute that response into Eq. (3) to evaluate the output field *E*(*z*, *t*).

## 3 Field solutions

In this section, we shall derive the general field solutions for the GRIN system in Figure 2(b) using the generalized impulse response derived in Section 2.

The refractive index profile along the impulse trajectory is given by

(8) $$n\left(z,t\right)=\left\{\begin{array}{cc} n_{1},\; & 0<z<z_{\text{b}1}^{0}+vt,\\ n_{\text{G}}\left(z-vt\right),\; & z_{\text{b}1}^{0}+vt<z<z_{\text{b}2}^{0}+vt,\\ n_{2},\; & z>z_{\text{b}2}^{0}+vt, \end{array}\right.$$

where *n*_1_ and *n*_2_ are the (constant) refractive indices of media 1 and 2, respectively, and

(9) $$z_{\text{b}2}^{0}=z_{\text{b}1}^{0}+D.$$

The system can then be divided into the three corresponding regions, which we will address one by one.

### 3.1 First-medium (*n*_1_) region

Substituting *n*(*z*, *t*_*n*_) = *n*_1_ into Eq. (6), we obtain the wave trajectory equation

(10a) $$c\frac{dt_{n}}{dz}=n_{1},$$

where the boundary condition is

(10b) $$t_{n}\left(0\right)=t^{\prime }.$$

Solving Eq. (10) for *t*_*n*_(*z*, *t*′) yields then the impulse trajectory function

(11) $$t_{n}\left(z,t^{\prime }\right)=t^{\prime }+\frac{n_{1}}{c}z,$$

whose insertion into Eq. (5) provides the impulse response

(12) $$h\left(z,t,t^{\prime }\right)=\delta \left(t-t^{\prime }-n_{1}\frac{z}{c}\right).$$

Note that the argument of the impulse function in this relation represents a *traveling-wave*, due to the uniform nature of the propagation medium. Finally, substituting Eq. (12) into (3) and solving for the output field *E*(*z*, *t*), we get the field,

(13) $$E_{1}\left(z,t\right)=E\left(0,t-n_{1}\frac{z}{c}\right),$$

where *E*(⋅, ⋅) is an arbitrary field function (e.g., harmonic plane wave or Gaussian pulse) of the space (first entry) and time (second entry) variables.3In Eq. (13), the bracket (⋅) indicates a functional argument. At other places in the paper, the argument may involve the square bracket [⋅] or the curl bracket {⋅}, because we use the bracket precedence order (⋅) − [⋅] − {⋅}. Whether the brackets indicate an argument or a multiplicative factor should be clear everywhere from the context.


### 3.2 GRIN (*n*_G_) region

Substituting now *n*(*z*, *t*_*n*_) = *n*_G_(*z* − *vt*_*n*_) into Eq. (6), we obtain the wave trajectory equation in the GRIN region,

(14) $$c\frac{dt_{n}}{dz}=n_{\text{G}}\left(z-vt_{n}\right).$$

The corresponding space–time boundary condition with medium 1 corresponds to the intersection point *p*_1_ [see Figure 2(b)], whose coordinates are related as

(15a) $$t_{\text{b}1}=t^{\prime }+\frac{n_{1}}{c}z_{\text{b}1}$$

and

(15b) $$z_{\text{b}1}=z_{\text{b}1}^{0}+vt_{\text{b}1}.$$

Solving Eq. (15) for *z*_b1_ and *t*_b1_, we find the coordinates of *p*_1_ to be

(16) $$z_{\text{b}1}=\frac{vt^{\prime }+z_{\text{b}1}^{0}}{1-n_{1}v/c}\;\text{and}\;t_{\text{b}1}=\frac{t^{\prime }+n_{1}z_{\text{b}1}^{0}/c}{1-n_{1}v/c}.$$

To solve Eq. (14) with the related boundary condition, we let *ξ* = *z* − *vt*_*n*_, which implies $\frac{d\xi }{dz}=1-v\frac{dt_{n}}{dz}$ , or

(17) $$\frac{dt_{n}}{dz}=\frac{1}{v}\left(1-\frac{d\xi }{dz}\right).$$

Substituting now Eq. (17) into (14), and separating the *ξ* and *z* terms, leads to the differential equation

(18) $$\frac{d\xi }{1-n_{\text{G}}\left(\xi \right)v/c}=dz,$$

which integrates to

(19) $$\int \frac{1}{1-n_{\text{G}}\left(\xi \right)v/c}\;d\xi =z+C_{G},$$

where *C*_G_ is an integration constant. For generalization to arbitrary GRIN profiles, we define the left-hand side term of this relation as the function

(20) $$F\left(\xi \right)=\int \frac{1}{1-n_{\text{G}}\left(\xi \right)v/c}\;d\xi ,$$

which allows to express Eq. (19) in the compact form

(21) $$F\left(\xi \right)=z+C_{G}.$$

To determine the integration constant *C*_G_ in this relation, we first apply the boundary condition *t*_*n*_(*z*_b1_) = *t*_b1_ (point *p*_1_), which yields

(22) $$F\left(z_{\text{b}1}-vt_{\text{b}1}\right)=z_{\text{b}1}+C_{G}.$$

Next substituting Eq. (16) into this relation and solve for *C*_G_, we obtain

(23) $$C_{G}\left(t^{\prime }\right)=-\frac{v}{1-n_{1}v/c}t^{\prime }-\frac{z_{\text{b}1}^{0}}{1-n_{1}v/c}+F\left(z_{\text{b}1}^{0}\right).$$

Finally, substituting Eq. (23) and the relation *ξ* = *z* − *vt*_*n*_ into Eq. (21), and solving for *t*_*n*_, we find

(24) $$t_{n}\left(z,t^{\prime }\right)=-\frac{1}{v}F^{-1}\left[z+C_{G}\left(t^{\prime }\right)\right]+\frac{z}{v},$$

where *F* ^−1^(⋅) is the inverse function of *F*(⋅).

Substituting Eq. (24) into (5), we obtain now the impulse response

(25) $$h\left(z,t,t^{\prime }\right)=\delta \left\{t+\frac{1}{v}F^{-1}\left[z+C_{G}\left(t^{\prime }\right)\right]-\frac{z}{v}\right\},$$

where *C*_G_(*t*′) is given in Eq. (23) and *F*(⋅) is defined in Eq. (20).

Finally, substituting Eq. (25) into (3), and solving for the output field *E*(*z*, *t*), yields (see Appendix A)

(26) $$\begin{array}{cc} E_{\text{G}}\left(z,t\right) & =\left|\frac{1-n_{1}v/c}{1-n_{\text{G}}\left(z-vt\right)v/c}\right|\\ & \;\times E\left[0,-\frac{1-n_{1}v/c}{v}\overset{z}{\underset{z_{\text{b}1}^{0}+vt}{\int }}\frac{1}{1-n_{\text{G}}\left(z^{\prime }-vt\right)v/c}\;dz^{\prime }\right.\\ & \;\left.-n_{1}\frac{z}{c}+\frac{z-z_{\text{b}1}^{0}}{v}\right], \end{array}$$

where *E*(⋅, ⋅) is the same field function of space and time as in Eq. (13).

### 3.3 Second-medium (*n*_2_) region

Substituting now *n*(*z*, *t*_*n*_) = *n*_2_ into Eq. (6), we obtain

(27) $$c\frac{dt_{n}}{dz}=n_{2},$$

where the space–time boundary condition with the GRIN medium corresponding to the intersection point *p*_2_, whose coordinates are related as

(28a) $$t_{\text{b}2}=-\frac{1}{v}F^{-1}\left[z_{\text{b}2}+C_{G}\left(t^{\prime }\right)\right]+\frac{z_{\text{b}2}}{v}$$

and

(28b) $$z_{\text{b}2}=z_{\text{b}2}^{0}+vt_{\text{b}2},$$

and found by solving Eq. (28) for *z*_b2_ and *t*_b2_ as

(29a) $$z_{\text{b}2}=F\left(z_{\text{b}2}^{0}\right)-C_{G}\left(t^{\prime }\right)$$

and

(29b) $$t_{\text{b}2}=\frac{F\left(z_{\text{b}2}^{0}\right)-C_{G}\left(t^{\prime }\right)-z_{\text{b}2}^{0}}{v}.$$

Solving next Eq. (27) for *t*_*n*_(*z*, *t*′), we obtain the impulse trajectory function

(30a) $$t_{n}\left(z,t^{\prime }\right)=\frac{n_{2}}{c}z+C_{2},$$

where *C*_2_ is a new integration constant, which is obtained by applying the boundary condition *t*_*n*_(*z*_b2_) = *t*_b2_ [Eq. (29), point *p*_2_] to Eq. (30a) and solving the resulting expression for *C*_2_ as

(30b) $$\begin{array}{ll} C_{2}\left(t^{\prime }\right) & =\frac{1-n_{2}v/c}{1-n_{1}v/c}t^{\prime }+\frac{1-n_{2}v/c}{v}\left[F\left(z_{\text{b}1}^{0}+D\right)\right.\\ & \;\left.-F\left(z_{\text{b}1}^{0}\right)\right]-\frac{n_{2}-n_{1}}{1-n_{1}v/c}\frac{z_{\text{b}1}^{0}}{c}-\frac{D}{v}. \end{array}$$

Finally, substituting Eq. (30) into (5), we get the impulse response

(31) $$h\left(z,t,t^{\prime }\right)=\delta \left[t-\frac{n_{2}}{c}z-C_{2}\left(t^{\prime }\right)\right],$$

where *C*_2_(*t*′) is given in Eq. (30b).

Substituting Eq. (31) into (3) and solving for the output field *E*(*z*, *t*) (see Appendix B), we obtain the field4In Eq. (32), the denominator of the factor $\frac{1-n_{1}v/c}{1-n_{2}v/c}$ vanishes when *v* = *c*/*n*_2_. This corresponds to one of the interluminal boundaries [20], where the interface moves at the same velocity as the wave in the second medium. In this regime, continuous “pushing” leads to extreme wave compression and amplification, ultimately forming a shock wave. Although theoretically valid, operation at modulation velocities close to *v* = *c*/*n*_2_ should be avoided in practice to prevent nonlinearity-induced distortion.

(32) $$\begin{array}{cc} E_{2}\left(z,t\right) & =\left|\frac{1-n_{1}v/c}{1-n_{2}v/c}\right|\\ & \;\times E\left\{0,\frac{1-n_{1}v/c}{1-n_{2}v/c}\left[t-\frac{n_{2}}{c}z+\frac{n_{2}-n_{1}}{1-n_{1}v/c}\frac{z_{\text{b}1}^{0}}{c}+\frac{D}{v}\right.\right.\\ & \;\left.\left.-\frac{1-n_{2}v/c}{v}\overset{z_{\text{b}1}^{0}+D+vt}{\underset{z_{\text{b}1}^{0}+vt}{\int }}\frac{1}{1-n_{\text{G}}\left(z^{\prime }-vt\right)v/c}\;dz^{\prime }\right]\right\}. \end{array}$$

Equations (13), (26), and (32) represent the key results of this paper. They accommodate arbitrary field waveforms *E*(0, *t*) and arbitrary GRIN profiles *n*_G_(*z* − *vt*).

## 4 Chirping physics

Due to the space- and time-varying properties of the GRIN medium, the wave behavior in our system is fairly complex, as evident in Eq. (26). In this section, we show that this system produces a new type of wave chirping and compare it with other chirping mechanisms.

### 4.1 Chirping in the GRIN layer

We consider a time-harmonic incident field, which reads at *z* = 0,

(33) $$E\left(0,t\right)=e^{-i\omega_{0}t},$$

where *ω*_0_ is assumed to be constant. Other types of fields can be treated similarly. Substituting Eq. (33) into (26) – specifically, inserting the content of the second slot of *E*(⋅, ⋅) in Eq. (26) into the relation *ϕ*_G_ = −*ω*_0_ *t*(*z*) – we obtain the wave phase in the GRIN layer as

(34) $$\begin{array}{cc} \phi_{\text{G}} & =\omega_{0}\frac{1-n_{1}v/c}{v}\overset{z}{\underset{z_{\text{b}1}^{0}+vt}{\int }}\frac{1}{1-n_{\text{G}}\left(z^{\prime }-vt\right)v/c}\;dz^{\prime }\\ & \;+\omega_{0}\left(n_{1}\frac{z}{c}-\frac{z-z_{\text{b}1}^{0}}{v}\right). \end{array}$$

The related instantaneous frequency at a given position in the GRIN layer is obtained by differentiating the phase in Eq. (34) with respect to time, yielding

(35) $$\begin{array}{cc} \omega_{\text{G}} & =-\omega_{0}\frac{1-n_{1}v/c}{v}\frac{\partial }{\partial t}\left[\overset{z}{\underset{z_{\text{b}1}^{0}+vt}{\int }}\frac{1}{1-n_{\text{G}}\left(z^{\prime }-vt\right)v/c}\;dz^{\prime }\right]\\ & =\omega_{0}\frac{1-n_{1}v/c}{1-n_{G}\left(z-vt\right)v/c}, \end{array}$$

where we have used the Leibniz integral rule. Equation (35) reveals that the wave frequency in the GRIN layer varies with time, through the function *n*_G_(*z* − *vt*) in the denominator, indicating a *space–time chirping effect*. This effect is fundamentally different from the group-velocity dispersion (GVD) chirping effect occurring in dispersive media [18], since no dispersion is present. It will be explained shortly.

Furthermore, the chirp parameter *α*, whose sign determines whether the field is up-chirping (*α* > 0) or down-chirping (*α* < 0), is obtained by time-differentiating the instantaneous frequency in Eq. (35), which gives

(36) $$\alpha =\frac{\partial \omega_{\text{G}}}{\partial t}=-\omega_{0}\frac{\left(1-n_{1}v/c\right)}{c{\left[1-n_{\text{G}}\left(z,t\right)v/c\right]}^{2}}\frac{\partial n_{\text{G}}\left(z,t\right)}{\partial z}.$$

This result indicates that the sign of the chirp, *α* ≷ 0, depends on

(37) $$\left(1-n_{1}v/c\right)\partial n_{\text{G}}\left(z,t\right)/\partial z≶0.$$

According to Eq. (37), the up- or down-chirping behavior of the system is governed by the velocity regime, which may be subluminal [*v* < *c*/max(*n*_1_, *n*_2_)] or superluminal [*v* > *c*/min(*n*_1_, *n*_2_)] [21]. To understand the chirping mechanism within the GRIN layer, we now focus on the subluminal case – the superluminal case can be analyzed analogously. In the subluminal regime, where *v* < *c*/*n*_1_,5When *n*_1_ > *n*_2_, the subluminal regime is strictly defined by *v* < *c*/*n*_1_. However, if *n*_1_ < *n*_2_, the subluminal regime becomes *v* < *c*/*n*_2_, which still falls within the range *v* < *c*/*n*_1_ since *c*/*n*_2_ < *c*/*n*_1_.
Eq. (37) simplifies to

(38) $$\partial n_{\text{G}}\left(z,t\right)/\partial z≶0,$$

indicating that a decreasing refractive index slope leads to an increase in the instantaneous frequency, i.e., up-chirping, and vice versa, i.e., down-chirping.

For simplicity, but without loss of generality, we consider the simplest GRIN profile, the linear profile.

(39) $$n_{G}\left(z-vt\right)=n_{1}+\frac{n_{2}-n_{1}}{D}\left(z-vt-z_{\text{b}1}^{0}\right).$$

**Figure 3:**
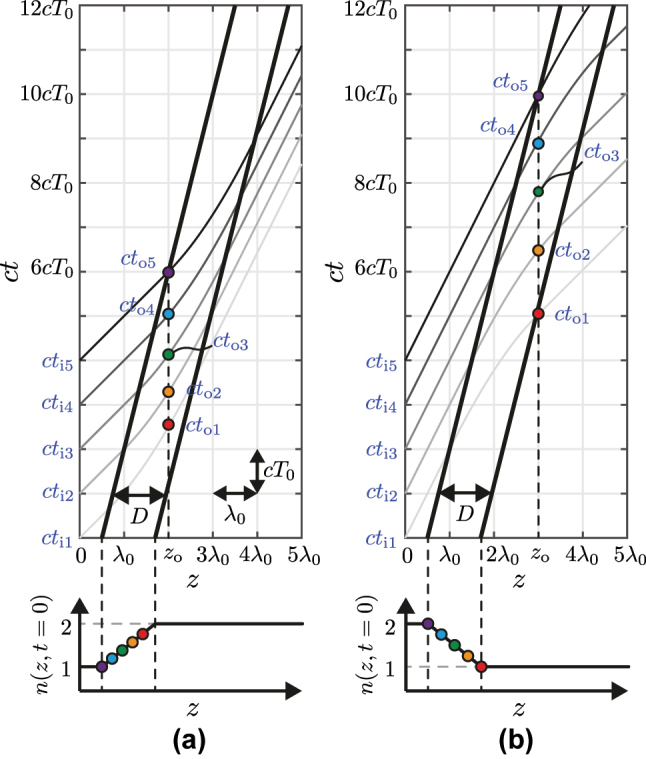
Space–time diagrams of a linearly varying GRIN medium [Eq. (39)] in the subluminal velocity regime (*v* = 0.25*c*) used for the chirping analysis, with (a) a positive *n*_G_ slope, where *n*_1_ = 1, *n*_2_ = 2 and *D* = 1.2*λ*_0_ with *λ*_0_ = *cT*_0_ being the free-space wavelength, and (b) a negative *n*_G_ slope, where *n*_1_ = 2, *n*_2_ = 1 and *D* = 1.2*λ*_0_. The bottom panels show the corresponding initial refractive index profiles, *n*(*z*, *t* = 0).

Figure 3 represents the corresponding trajectories for the wave crests incident at *z* = 0 at the times *t*_i1,2,3,4,5_, as derived from the analytical solutions in the different regions, given by Eqs. (13), (26), and (32). Figure 3(a) considers the case where the refractive index increases within the GRIN layer, i.e., *n*_2_ > *n*_1_, corresponding to the positive spatial gradient *∂n*_G_(*z*, *t*)/*∂z* > 0, which leads to a down-chirping according to the condition in Eq. (38). For a fixed observation position *z*_o_ within the GRIN region, the arrival times of the five incident crests are denoted as *t*_o1,2,3,4,5_. Due to the space–time variation of *n*_G_, each crest experiences a different local refractive index at the observation point, as indicated by the color-coded dots in the figure. The latest crest (purple dot) propagates fastest, as it has just entered the GRIN region and hence encounters the lowest refractive index. In contrast, the earliest crest (red dot) travels slowest, as it is near the exit of the GRIN region and hence sees the highest refractive index. As a result, the later, faster crest gradually catches up with the earlier, slower one, leading to an increasing temporal separation between adjacent crests. This corresponds to a decreasing frequency over time, i.e., down-chirping, consistent with the down-chirping condition in Eq. (38). A similar mechanism applies in the case of a decreasing refractive index, i.e., *n*_2_ < *n*_1_, where the crests compress in time, resulting in up-chirping [Eq. (38)], as illustrated in Figure 3(b).

Note that the wave exiting the GRIN layer is no longer chirped. This because, at the exit points, all the crest have experienced the entire GRIN profile and find, therefore, themselves resynchronized to the velocity of the second medium.

The relation (35) offers a practical foundation for designing systems with a prescribed chirp profile, where the frequency varies according to a desired function *f*(*t*),

(40) $$\omega_{G}=f\left(t\right).$$

To realize such a frequency evolution, one can tailor the refractive index profile of the GRIN layer, by substituting Eq. (35) into (40), and solving for *n*_G_, yielding

(41) $$n_{\text{G}}=\frac{c}{v}\left[1-\frac{\omega_{0}\left(1-n_{1}v/c\right)}{f\left(t\right)}\right],$$

which provides a closed-form expression for engineering a GRIN profile generating the desired chirp. As an example, for a linear chirp, where

(42) f(t)=a+b(z−vt)

with *a* and *b* being constants, the refractive index profile becomes

(43) $$n_{\text{G}}=\frac{c}{v}\left[1-\frac{\omega_{0}\left(1-n_{1}v/c\right)}{a+b\left(z-vt\right)}\right].$$

### 4.2 Comparison with other chirping mechanisms

In this section, we review the main chirping mechanisms and compare them with the GRIN-based mechanism described in the previous section.

*Dispersive systems* are the most common conventional means of generating chirping. In these systems, group-velocity dispersion (GVD) [18] causes a frequency-dependent group delay, resulting in a quadratic (or higher-order) spectral phase and corresponding variations in the instantaneous frequency across the pulse. Since this process alters only the spectral phase while leaving the spectral amplitude unchanged, the temporal waveform is scaled without spectral alteration, which limits the achievable chirp range for a given input bandwidth. Chirping can also arise in *nonlinear systems* [22]. A common example is self-phase modulation (SPM) [18], in which a high-intensity pulse propagating through a Kerr medium experiences an intensity-dependent refractive index change, imprinting a corresponding nonlinear phase on the pulse. The resulting time-varying phase produces instantaneous frequency shifts that evolve along the propagation distance. Because the effect scales nonlinearly with the optical intensity and interaction length, SPM-induced chirping offers limited independent control over the chirp profile and is less straightforward to tune than dispersive methods.

Recently, other chirping mechanisms have been explored in LTV systems. In these structures, chirping arises from various effects at a time-varying interface, which generate new frequency components and thereby alters the magnitude spectrum – in both shape and bandwidth – within the linear regime. For instance, Shlivinski and Hadad demonstrated transient chirped-like radiation from a *lossy, dispersive time-varying slab* into air, attributed to the conservation of longitudinal wavenumber at the temporal interface [23]. Another example involves *accelerated space–time step-index interfaces* [24], [25], [26], where nonuniform modulation velocities induce Doppler-based time-dependent frequency transitions and associated chirping effects.

The space–time GRIN-based chirping system presented in this paper represents a new class of LTV chirping mechanisms. In these systems, chirping arises from the space–time variation of medium properties within the modulation slab, which induces local wave velocity differences that modify the instantaneous frequency. Compared to the other two LTV structures, GRIN-based systems offer greater design flexibility for chirping and do not require complicated nonuniform modulation velocities.

## 5 Illustrative results

**Figure 4:**
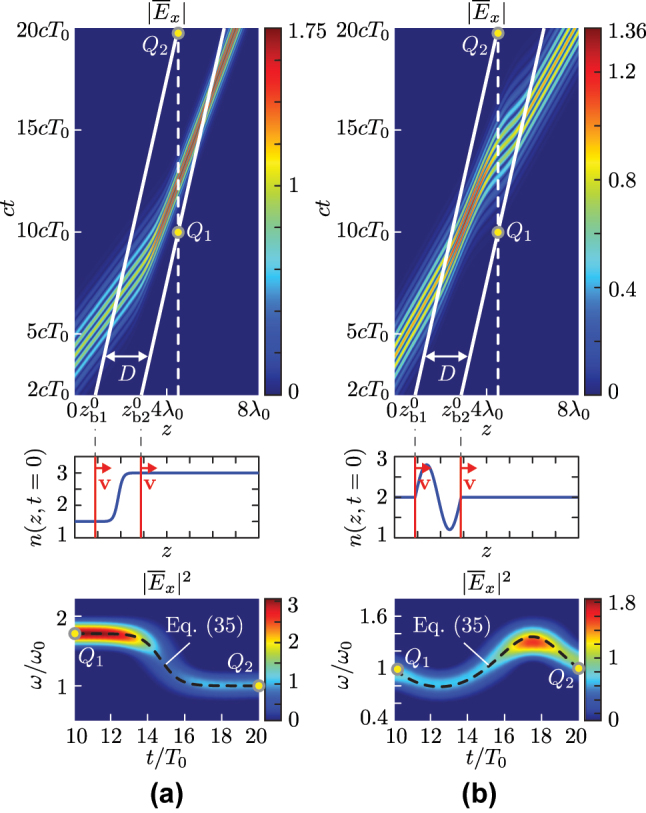
Electric field magnitude |*E*_*x*_| across space–time GRIN interfaces, computed by Eqs. (13), (26), and (32), for the input pulse $E\left(0,ct\right)=e^{-{\left(t-4T_{0}\right)}^{2}/2T_{0}^{2}}e^{-i\omega_{0}t}$ , interface velocity *v* = 0.2*c*, and different GRIN profiles, (a) a hyperbolic tangent profile, $n_{G}\left(z-vt\right)=n_{1}+\left[\left(n_{2}-n_{1}\right)/2\right]\left[1+10\tanh\left(z-vt-z_{\text{b}1}^{0}-D/2\right)/D\right]$ , with *n*_1_ = 1.5, *n*_2_ = 3 and *D* = 2*λ*_0_, and (b) a sine profile, $n_{G}\left(z-vt\right)=n_{1}+\left[0.8+n_{2}-n_{1}\right]\sin\left[2\pi \left(z-vt-z_{\text{b}1}^{0}\right)/D\right]$ , with *n*_1_ = *n*_2_ = 2 and *D* = 2*λ*_0_. The top panels show the space–time diagrams of the normalized electric field magnitude $\left[|{\bar{E}}_{x}\left(z,ct\right)|=|E_{x}\left(z,ct\right)|/\max\left(\left|E_{x}\left(0,ct\right)\right|\right)\right]$ under modulated Gaussian pulse excitation, where the white solid lines mark the two boundaries of the GRIN region. The middle panels show the refractive index profiles *n*(*z*, *t*) [Eq. (8)] at *t* = 0. The bottom panels show the normalized spectrograms, $|{\bar{E}}_{x}\left(t,\omega \right){|}^{2}$ , with the input pulse being replaced by the quasi-continuous wave $E\left(0,t\right)=e^{-i\omega_{0}t}\text{rect}\left(t/\tau \right)$ (with *τ* = 30*T*_0_), for easier visualization, across the GRIN layer at *z* = 4.5*λ*_0_ between points *Q*_1_ and *Q*_2_. The dashed black line corresponds to the instantaneous frequency *ω*_G_(*t*) given by Eq. (35).

Figure 4 plots the electric field magnitudes across two space–time GRIN interfaces computed by Eqs. (13), (26), and (32). Figure 4(a) corresponds to a hyperbolic tangent GRIN interface profile. It may be observed that the field experiences a gradual down-chirping in the GRIN region, as expected from *α* < 0 [Eq. (38)], before reaching a steady frequency in the second medium. Figure 4(b) corresponds to a sinusoidal GRIN interface profile. In this case, the field undergoes nonmonotonic, twisted chirping within the GRIN layer with varying alternating chirping sign [Eq. (38)], and eventually recovers its original frequency after exiting the modulated region. In both cases, the closed-form field distributions in the figure have been validated against full-wave finite-difference time-domain (FDTD) simulations [15], [24] (see Appendix C).

**Figure 5:**
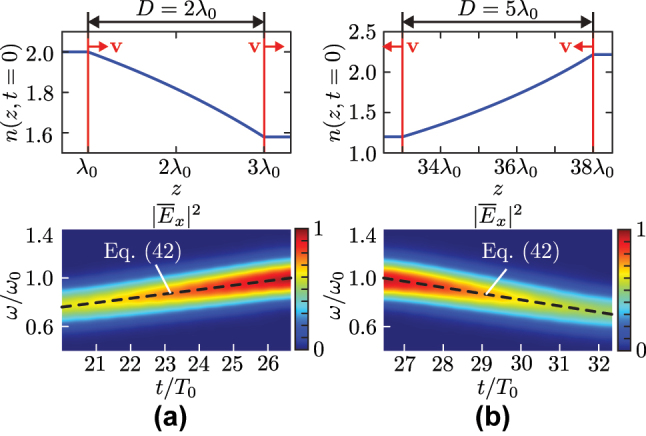
Design of a linear-chirping [Eq. (42)] GRIN system, corresponding to the refractive index profiles obtained from Eq. (43) and shown in the top panels. The input pulse is the same quasi-continuous wave as in the bottom panels of Figure 4. (a) Up-chirping system with *n*_1_ = 2, *n*_2_ = 1.58, *v* = 0.3*c* (subluminal regime), *D* = 2*λ*_0_, *a* = 1 and *b* = −0.4. (b) Down-chirping system with *n*_1_ = 1.2, *n*_2_ = 2.22, *v* = −0.85*c* (superluminal regime), *D* = 5*λ*_0_, *a* = 1 and *b* = −0.2. The top and bottom panels show the GRIN profiles at *t* = 0 and the corresponding spectrograms, $|{\bar{E}}_{x}\left(t,\omega \right){|}^{2}$ , respectively. The dashed black lines represent the target linear-chirp function *f*(*t*) [Eq. (42)].

Figure 5 presents two linear-chirping GRIN designs using Eq. (43). In Figure 5(a), the design is performed in a co-moving (up-chirping) subluminal regime with *v* = 0.3*c*, while in Figure 5(b), it corresponds to a contra-moving (down-chirping) superluminal regime with *v* = −0.85*c*. The spectrograms in the bottom panels, corresponding to the GRIN profiles in the top panels, precisely match the theoretical predictions (see Appendix C).

## 6 Conclusion and discussion

We have presented an exact electromagnetic solution to the problem of wave propagation across arbitrary space–time modulated GRIN interfaces and a detailed description of the related chirping effects. This solution offers a novel approach to chirp generation, which can be realized using artificial transmission lines at microwave frequencies [27], [28], [29] and acoustic or optical wave-based modulation techniques at optical frequencies [8], [9], [10], [30].

In the microwave regime, such interfaces can be realized in the subluminal regime by injecting pump pulses into transmission lines loaded with nonlinear capacitive and inductive elements, such as varactors and ferrite cores [28]. The pump signal applied at the terminal propagates along the line, inducing a dynamic, intensity-dependent refractive index variation that forms a moving GRIN interface between regions with different electromagnetic properties. A linear probe signal interacting with this modulation interface undergoes the designed chirping effect. While achieving superluminal modulation velocities is impossible with such a pump-probe platform, it could be potentially realized using switched transmission lines composed of subwavelength units spaced by Δ*z*, each loaded with a sequence of elements having different parameters controlled by switches to create an effective spatial gradient, *n*_G_(*z*). An external controller, such as a field-programmable gate array (FPGA), actuates these switches with a time interval Δ*t*, enabling sequential time delays [27]. By adjusting the ratio Δ*z*/Δ*t*, this spatial gradient can propagate at an effectively unlimited velocity *v* = Δ*z*/Δ*t* ∈ (0, ∞), forming an effective moving GRIN interface, *n*_G_(*z* − *vt*).

In the optical regime, similar pump–probe setup can be achieved by launching a strong pump obliquely at an angle *θ* relative to the probe, producing a modulation velocity *v* = *c*/sin*θ* ∈ (0, ∞) that spans both subluminal and superluminal regimes [30]. Dynamic permittivity modulation can be realized via surface or bulk acoustic waves in piezoelectric crystals, ultrafast laser pulses in semiconductor slabs, or epsilon-near-zero (ENZ) materials for higher refractive index contrast [10]. Dynamic control of permeability can be achieved through the magneto-optical response of gyromagnetic materials. Experimental challenges in realizing arbitrary GRIN profiles may be addressed by employing arbitrary waveform generators, enabling synthesis of the desired modulation profiles with high precision. This paper advances the modeling of space–time dispersive systems and introduces a new paradigm for linear pulse shaping. The approach can also be integrated with various platforms for dispersion compensation, enabling enhanced control over wave propagation in dynamic media.

## Figures and Tables

**Figure 6:**
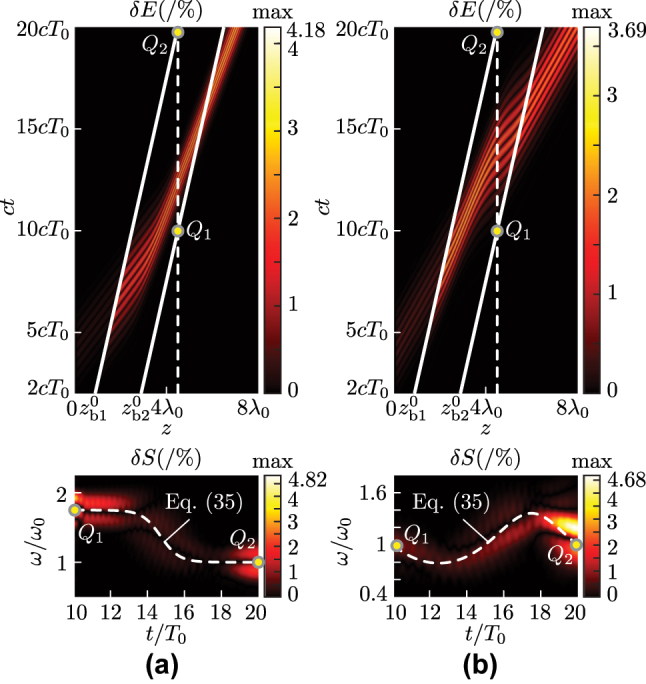
Difference between the exact theoretical results in Figure 4 and full-wave FDTD results, with difference attributed to simulation (meshing) approximation errors.

**Figure 7:**
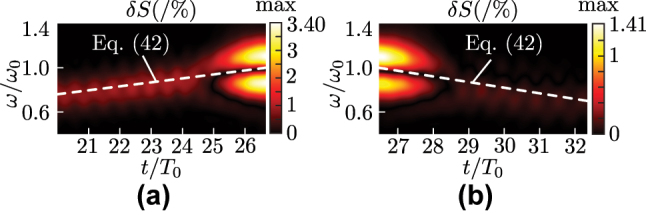
Same as in Figure 6 but for Figure 5.

## Appendix A Derivation of Eq. (26)

In this appendix, we provide a detailed derivation of Eq. (26) given in Section 3.2. Substituting Eq. (25) into (3), we get

(44)
$$\begin{array}{cc} & E_{\text{G}}\left(z,t\right)\\ & \;=\overset{\infty }{\underset{-\infty }{\int }}\delta \left\{t+\frac{1}{v}F^{-1}\left[z+C_{G}\left(t^{\prime }\right)\right]-\frac{z}{v}\right\}E\left(0,t^{\prime }\right)\;dt^{\prime }. \end{array}$$

To simplify the notation, we define the argument of the delta function in Eq. (44) as

(45)
$$g_{G}\left(t^{\prime }\right)=t+\frac{1}{v}F^{-1}\left[z+C_{G}\left(t^{\prime }\right)\right]-\frac{z}{v}.$$

Moreover, we use the following property of the delta function [31]

(46)
$$\overset{\infty }{\underset{-\infty }{\int }}\delta \left[g_{G}\left(t^{\prime }\right)\right]f\left(0,t^{\prime }\right)\;dt^{\prime }=\frac{f\left(0,t_{G}^{\prime }\right)}{\left|g_{G}^{\prime }\left(t_{G}^{\prime }\right)\right|},$$

where $g_{G}\left(t_{G}^{\prime }\right)=0$ and $g_{G}^{\prime }\left(\cdot \right)=dg_{G}\left(\cdot \right)/dt^{\prime }$ . Therefore, we first solve for the root $t_{G}^{\prime }$ of *g*_G_(*t*′) = 0, i.e.,

(47)
$$t+\frac{1}{v}F^{-1}\left[z+C_{G}\left(t_{G}^{\prime }\right)\right]-\frac{z}{v}=0,$$

which gives

(48)
$$C_{G}\left(t_{G}^{\prime }\right)=F\left(\xi \right)-z.$$

Substituting Eq. (23) into the left-hand side of Eq. (48) and solving for $t_{G}^{\prime }$ , we find

(49)
$$t_{G}^{\prime }=-\frac{1-n_{1}v/c}{v}\left[F\left(\xi \right)-F\left(z_{\text{b}1}^{0}\right)\right]-n_{1}\frac{z}{c}+\frac{z-z_{\text{b}1}^{0}}{v}.$$

Applying Eq. (20) and performing the change of variables *z*′ = *ξ* + *vt*, the difference in the square brackets of Eq. (49) can be written as

(50)
$$F\left(\xi \right)-F\left(z_{\text{b}1}^{0}\right)=\overset{z}{\underset{z_{\text{b}1}^{0}+vt}{\int }}\frac{1}{1-n_{\text{G}}\left(z^{\prime }-vt\right)v/c}\;dz^{\prime }.$$

Substituting Eq. (50) into (49) yields the final expression

(51)
$$\begin{array}{cc} t_{G}^{\prime } & =-\frac{1-n_{1}v/c}{v}\overset{z}{\underset{z_{\text{b}1}^{0}+vt}{\int }}\frac{1}{1-n_{\text{G}}\left(z^{\prime }-vt\right)v/c}\;dz^{\prime }\\ & \;-n_{1}\frac{z}{c}+\frac{z-z_{\text{b}1}^{0}}{v}. \end{array}$$

Next, we determine the derivative of *g*_G_(*t*′) [Eq. (45)] with respect to *t*′ by applying the chain rule and introducing the substitution *u* = *z* + *C*_G_(*t*′), yielding

(52)
$$\frac{dg_{G}\left(t^{\prime }\right)}{dt^{\prime }}=\frac{1}{v}\frac{dF^{-1}\left(u\right)}{du}\frac{dC_{G}\left(t^{\prime }\right)}{dt^{\prime }}.$$

Let *w* = *F* ^−1^(*u*), i.e., *u* = *F*(*w*). Then the derivative d*F* ^−1^(*u*)/d*u* on the right-hand side of Eq. (52) can be expressed as

(53)
$$\frac{dF^{-1}\left(u\right)}{du}=\frac{dw}{du}=\frac{1}{\frac{du}{dw}}=\frac{1}{F^{\prime }\left(w\right)}=\frac{1}{F^{\prime }\left[F^{-1}\left(u\right)\right]},$$

where *F*′(⋅) denotes the derivative of *F* with respect to its argument. Using Eq. (23), the derivative d*C*_G_(*t*′)/d*t*′ on the right-hand side of Eq. (52) is given by

(54)
$$\frac{dC_{G}\left(t^{\prime }\right)}{dt^{\prime }}=-\frac{v}{1-n_{1}v/c}.$$

Substituting Eqs. (53) and (54) into (52), we obtain

(55)
$$\frac{dg_{G}\left(t^{\prime }\right)}{dt^{\prime }}=-\frac{1}{1-n_{1}v/c}\frac{1}{F^{\prime }\left[F^{-1}\left(u\right)\right]}.$$

At the root $t_{G}^{\prime }$ , using Eq. (47), we find

(56)
$$F^{-1}\left[u\left(t_{G}^{\prime }\right)\right]=z-vt=\xi .$$

Then, applying the definition in Eq. (20), we get

(57)
$$F^{\prime }\left\{F^{-1}\left[u\left(t_{G}^{\prime }\right)\right]\right\}=F^{\prime }\left(\xi \right)=\frac{1}{1-n_{\text{G}}\left(\xi \right)v/c}.$$

Substituting Eq. (57) into (55) and using the relation *ξ* = *z* − *vt*, we find the derivative of *g*_G_(*t*′) evaluated at the root

(58)
$${\left.\frac{dg_{G}\left(t^{\prime }\right)}{dt^{\prime }}\right|}_{t_{G}^{\prime }}=-\frac{1-n_{\text{G}}\left(z-vt\right)v/c}{1-n_{1}v/c}.$$

Finally, substituting Eqs. (45), (51), and (58), along with the relation *f*(0, *t*′) = *E*(0, *t*′), into the delta function identity [Eq. (46)], we evaluate the integral in Eq. (44) and find the result in Eq. (26).

## Appendix B Derivation of Eq. (32)

In this appendix, we provide a detailed derivation of Eq. (32) given in Section 3.3. Substituting Eq. (31) into (3), we get

(59)
$$E_{2}\left(z,t\right)=\overset{\infty }{\underset{-\infty }{\int }}\delta \left[t-\frac{n_{2}}{c}z-C_{2}\left(t^{\prime }\right)\right]E\left(0,t^{\prime }\right)\;dt^{\prime }.$$

We define the argument of the delta function in Eq. (59) as

(60)
$$g_{2}\left(t^{\prime }\right)=t-\frac{n_{2}}{c}z-C_{2}\left(t^{\prime }\right),$$

where *C*_2_(*t*′) is given in Eq. (30b).

To evaluate the convolution in Eq. (59), we use again the property [31]

(61)
$$\overset{\infty }{\underset{-\infty }{\int }}\delta \left[g_{2}\left(t^{\prime }\right)\right]f\left(0,t^{\prime }\right)\;dt^{\prime }=\frac{f\left(0,t_{2}^{\prime }\right)}{\left|g_{2}^{\prime }\left(t_{2}^{\prime }\right)\right|},$$

where $g_{2}\left(t_{2}^{\prime }\right)=0$ and $g_{2}^{\prime }\left(\cdot \right)=dg_{2}\left(\cdot \right)/dt^{\prime }$ . Substituting Eq. (30b) into (59) and solving *g*_2_(*t*′) = 0 for $t_{2}^{\prime }$ , we obtain

(62)
$$\begin{array}{cc} t_{2}^{\prime } & =\frac{1-n_{1}v/c}{1-n_{2}v/c}\left\{t-\frac{n_{2}}{c}z+\frac{n_{2}-n_{1}}{1-n_{1}v/c}\frac{z_{\text{b}1}^{0}}{c}+\frac{D}{v}\right.\\ & \;\left.-\frac{1-n_{2}v/c}{v}\left[F\left(z_{\text{b}1}^{0}+D\right)-F\left(z_{\text{b}1}^{0}\right)\right]\right\}. \end{array}$$

Applying Eq. (20) and performing the substitution *z*′ = *ξ* + *vt*, the difference in the square brackets of Eq. (62) can be written as

(63)
$$F\left(z_{\text{b}1}^{0}+D\right)-F\left(z_{\text{b}1}^{0}\right)=\overset{z_{\text{b}1}^{0}+D+vt}{\underset{z_{\text{b}1}^{0}+vt}{\int }}\frac{1}{1-n_{\text{G}}\left(z^{\prime }-vt\right)v/c}\;dz^{\prime }.$$

Substituting Eq. (63) into (62) yields the final expression

(64)
$$\begin{array}{cc} t_{2}^{\prime } & =\frac{1-n_{1}v/c}{1-n_{2}v/c}\left[t-\frac{n_{2}}{c}z+\frac{n_{2}-n_{1}}{1-n_{1}v/c}\frac{z_{\text{b}1}^{0}}{c}+\frac{D}{v}\right.\\ & \;\left.-\frac{1-n_{2}v/c}{v}\overset{z_{\text{b}1}^{0}+D+vt}{\underset{z_{\text{b}1}^{0}+vt}{\int }}\frac{1}{1-n_{\text{G}}\left(z^{\prime }-vt\right)v/c}\;dz^{\prime }\right]. \end{array}$$

The derivative of *g*_2_(*t*′) [Eq. (60)] with respect to *t*′, evaluated at the root, is then found as

(65)
$${\left.\frac{dg_{2}\left(t^{\prime }\right)}{dt^{\prime }}\right|}_{t_{2}^{\prime }}=\frac{dg_{2}\left(t^{\prime }\right)}{dt^{\prime }}=-\frac{1-n_{2}v/c}{1-n_{1}v/c}.$$

Finally, substituting Eqs. (60), (64), and (65), along with the relation *f*(0, *t*′) = *E*(0, *t*′), into the delta function identity [Eq. (61)], we evaluate the integral in Eq. (59) and find the result in Eq. (32).

## Appendix C FDTD validation

Figure 6 compares the results obtained by the theory in Figure 4 and by the FDTD method presented in [15], [24]. Specifically, it plots the field (top panels) and spectrogram (bottom panels) differences

(66a)
$$\delta E=\frac{\left|E_{x}-E_{\text{FDTD}}\right|}{\max\left(E_{x}\right)}\times 100\;\%$$

and

(66b)
$$\delta S=\left|\frac{|E_{x}{|}^{2}-|E_{\text{FDTD}}{|}^{2}}{\max\left(|E_{x}{|}^{2}\right)}\right|\times 100\;\%,$$

between the theoretical and simulation results, where *E*_*x*_ corresponds to the theoretical result and *E*_FDTD_ corresponds to the simulation result. As shown in Figure 6, the theoretical results closely match the simulation results (less than 5 % difference). This qualitatively validates the theory, while the fact that the theoretical results are exact (no approximation) suggests that the difference is due to the simulation (meshing) approximation errors, as we could verify by decreasing the mesh size up to the memory capability of our computer.

Figure 7 shows the corresponding difference for the designed linear-chirping GRIN interfaces shown in Figure 5, leading to the same conclusion.
